# Early-life stress does not alter spatial memory performance, hippocampal neurogenesis, neuroinflammation, or telomere length in 20-month-old male mice

**DOI:** 10.1016/j.ynstr.2021.100379

**Published:** 2021-08-12

**Authors:** Janssen M. Kotah, Lianne Hoeijmakers, Erik Nutma, Paul J. Lucassen, Aniko Korosi

**Affiliations:** Brain Plasticity Group, Center for Neuroscience, Swammerdam Institute for Life Sciences, University of Amsterdam, Science Park 904, Amsterdam, the Netherlands

**Keywords:** Early-life stress, Aging, Cognition, Neuroplasticity, Telomeres, ES, early-life stress, P, postnatal day, OF, open field test, MWM, Morris water maze, Tq, target quadrant, AU, aging-unimpaired, AI, aging impaired, DCX, doublecortin

## Abstract

Early-life stress (ES) increases the risk for psychopathology and cognitive decline later in life. Because the neurobiological substrates affected by ES (i.e., cognition, neuroplasticity, and neuroinflammation) are also altered in aging, we set out to investigate if and how ES in the first week of life affects these domains at an advanced age, and how ES modulates the aging trajectory per se. We subjected C57BL/6j mice to an established ES mouse model from postnatal days 2–9. Mice underwent behavioral testing at 19 months of age and were sacrificed at 20 months to investigate their physiology, hippocampal neuroplasticity, neuroinflammation, and telomere length. ES mice, as a group, did not perform differently from controls in the open field or Morris water maze (MWM). Hippocampal neurogenesis and synaptic marker gene expression were not different in ES mice at this age. While we find aging-associated alterations to neuroinflammatory gene expression and telomere length, these were unaffected by ES. When integrating the current data with those from our previously reported 4- and 10-month-old cohorts, we conclude that ES leads to a ‘premature’ shift in the aging trajectory, consisting of early changes that do not further worsen at the advanced age of 20 months. This could be explained e.g. by a ‘floor’ effect in ES-induced impairments, and/or age-induced impairments in control mice. Future studies should help understand how exactly ES affects the overall aging trajectory.

## Highlights

• Early-life stress (ES) exposure does not worsen water maze learning in aged male mice.
• ES does not affect brain plasticity markers at 20 months of age.
• Hippocampal telomere length is reduced by aging but unaffected by ES.
• ES leads to a premature aging trajectory that does not worsen with aging.

## 1 Introduction

Individuals with a history of early-life stress (ES) are at increased risk to develop psychopathologies and negative mental health outcomes later in life (Bellis et al., 2019; Ferraro et al., 2016; Hughes et al., 2017; Schafer and Ferraro, 2012). ES-exposed individuals also exhibit a higher prevalence of mild cognitive impairment, which seems to be more severe in nature (Kang et al., 2017; Wang et al., 2016), and is accompanied by reductions in their hippocampal volume (Ahmed-Leitao et al., 2016; Riem et al., 2015; Teicher et al., 2012).

Similarly, pre-clinical models of ES also present with later impairments in cognition, particularly in hippocampal-dependent, spatial learning tasks (e.g. Morris water maze, object location), as confirmed in a recent meta-analysis of rodent ES models between 2–12 months of age (Bonapersona et al., 2019). In line with human data, these impairments are accompanied by decreases in hippocampal volume (Hoeijmakers et al., 2017; Naninck et al., 2015) and alterations in various forms of hippocampal neuronal plasticity (i.e. reductions in dendritic complexity, spine numbers and hippocampal neurogenesis) (Ivy et al, 2008, 2010; Naninck et al., 2015; Rice et al., 2008; Solas et al., 2010; Wang et al., 2013), as well as by an altered microglial profile (Hoeijmakers et al., 2017; Ślusarczyk et al., 2015).

It has been postulated that ES might interact with the aging process (Miller et al., 2011), and, indeed, aging affects many of the systems that are also targeted by ES. Aging rodents also typically exhibit cognitive decline that varies between individuals (Drapeau et al., 2003), and is associated with a reduced hippocampal volume, altered neuroplasticity, neurogenesis and synaptic plasticity (Drapeau et al., 2003; Foster, 1999; Kempermann, 2015; Lynch et al., 2006; Montaron et al., 2020; Rosenzweig and Barnes, 2003). In addition, the aging brain is characterized by neuroimmune dysregulation, as microglia shift to a pro-inflammatory state, under both baseline and challenged conditions (Barrientos et al., 2015; Matt and Johnson, 2016; Hickman et al., 2013; Raj et al., 2015). Finally, aging is characterized by telomere shortening, both in peripheral tissues as well as in the brain (Ain et al., 2018; Flanary and Streit, 2003).

Because of this overlap between systems affected by ES and aging, it is plausible to assume that ES would affect aging-related processes. Interestingly, it has been suggested that ES leads to a steeper age-related cognitive decline in rodents (Jauregui-Huerta et al., 2015; Solas et al., 2010; Vallée et al., 1999), but also that ES rather modulates individual variability, resulting in increased subpopulations of impaired and unimpaired learners (Oitzl et al., 2000). However, the trajectory of the emergence of ES-associated phenotypes across the lifespan, as well as how these phenotypes progress through advanced ages, remains unclear.

Here, we tested whether exposure of mice to ES impacts various measures at 20 months of age, including: i) physiological measures (i.e. body, adrenal gland, and thymus weights, as well as basal and peak corticosterone levels), ii) cognitive performance, iii) neuroplasticity (i.e. adult neurogenesis and synapse-related gene expression), iv) age-associated inflammatory gene expression, and v) telomere length in the hippocampus. Lastly, we compared the current data from 20-month-old mice to earlier collected (and partly reported) data from separate 4- and 10-month-old cohorts of ES exposed mice (Hoeijmakers et al, 2017, 2018a).

## 2 Materials and methods

### 2.1 Early-life stress paradigm and housing

**Fig. 1:**
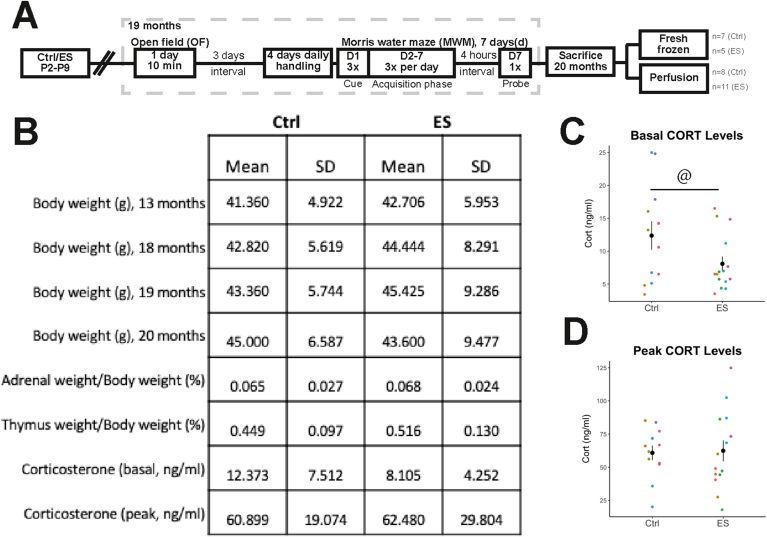
Physiological measures were not altered by ES. A) Experimental design and timeline of behavioral experiments. B) Table summarizing physiological measures in Ctrl and ES mice. C) ES leads to decreased variance in basal CORT levels at 20 months. D) Peak CORT levels are not affected by ES at 20 months. @, significant difference in variance, p < 0.05.

The experimental design is shown in Fig. 1A. C57Bl/6J male mice were bred in house to standardize their perinatal environmental conditions. Experimental breeding and the early-life stress (ES) paradigm were performed as described previously (Hoeijmakers et al., 2017; Naninck et al., 2015). Briefly, dams and pups were weighed on postnatal day (P) 2 and randomly placed in a control (Ctrl) cage with standard sawdust bedding and sufficient cotton nesting material (5 × 5cm, Technilab-BMI, The Netherlands), or in an early-life stress condition (ES) cage with reduced sawdust bedding, half the amount of nesting material, and a fine-gauge stainless steel mesh raised 1 cm above the cage floor. Dams and pups were left undisturbed until P9, when they were weighed and placed in standard cages until weaning at P21. Nests used had a minimum of 5 and a maximum of 6 pups, and always consisted of at least one female pup. Around 2–3 male pups were used per nest for the experimental procedures.

For the 20 month-old cohort, 15 Ctrl (from 6 litters) and 18 ES (from 8 litters) mice were included in the study. An additional cohort of Ctrl and ES mice at 4 and 10 months of age (n = 4 per group) were included for molecular analyses to assess age-related changes to gene expression and telomere length. Mice were housed with two to four littermates per cage under standard housing conditions, defined as a temperature of 20–22 °C, 40–60% humidity, cage enrichment and ad libitum standard chow and water. The mice were kept on a standard 12/12 h light/dark schedule (lights on at 8 a.m.) until 16 months of age and then transferred to a reversed 12/12 h light/dark schedule (lights on at 8 p.m.) to allow for a long period of acclimatization prior to behavioral analysis at 19 months of age. Body weight was monitored at 13, 18, 19 and 20 months of age.

2 out of the 33 males were excluded before the end of the study: one ES male was sacrificed at 18 months with a severe back lesion, one ES male died prematurely of an unknown cause. Experimental procedures were conducted according to the Dutch national law and European Union directives on animal experiments and were approved by the animal welfare committee of the University of Amsterdam.

### 2.2 Behavioral analysis

19-month-old mice were tested for basal locomotion and anxiety-related behavior in the open field (OF) and in the Morris water maze (MWM) for cognitive spatial performance (see Fig. 1A for design of behavioral experiments). Testing was conducted during the dark phase, with the testing room being lit by three 25 W red-light bulbs. To habituate, the mice were transferred to the room either 4 h (OF) or 1 h (MWM) prior to testing (Naninck et al., 2015). Performance was recorded and tracked for automated analysis of locomotion, velocity and position using Ethovision software (Noldus, The Netherlands).

#### 2.2.1 Open field

Mice were placed in a rectangular arena (54 × 37 × 33 cm) filled with sawdust bedding for 10 min in the dark with a red light on. The arena was cleaned in-between trials with 25% ethanol and half the amount of sawdust was replaced. The middle part of the arena (27 × 18.5 cm) was assigned as the center zone. Distance moved, locomotion, velocity and time in the center zone were analyzed.

#### 2.2.2 Morris water maze

Mice were handled by being picked up and allowed to walk along the experimenter's coat for 2 min for 4 consecutive days prior to the MWM. The protocol included 3 cued trials on day 1 (60 s trials; 15 min inter-trial time), followed by 3 daily acquisition trials (60 s trials; 15 min inter-trial time) for 6 consecutive days, and finally a single 60 sec-probe trial 4 h after the last acquisition trial (Kohman et al., 2013; van Praag et al., 2005). For all trials, mice were placed in the MWM pool at different, random starting positions to exclude ego-centric learning strategies and were placed in front of an infrared (heating) lamp for ±1 min after the trial to prevent hypothermia before being returned to their home cages.

For cued trials, mice were place in the circular MWM pool (110 cm in diameter), filled with clear water (24 ± 1 °C), and a visible 12 cm diameter, circular platform in the middle of the maze. Mice needed to reach the platform within 60 s, and were otherwise guided to the platform and placed on top for 15 s. For subsequent acquisition trials, the pool was surrounded by spatial cues to support allocentric spatial navigation. The water in the maze was made opaque using non-toxic paint, and the platform was submerged just below the water surface in a fixed position within the target quadrant (Tq). During the probe trial, the platform was removed from the Tq. Escape latency, swim path and velocity were analyzed for each of the trials.

### 2.3 Corticosterone measurements

Two days prior to sacrifice, tail blood was collected in EDTA-coated tubes with a tail cut at the beginning of the light-phase (08:00–08:15 a.m.) and at the beginning of the dark-phase (08:00–08:15 p.m.) for analysis of circulating basal and peak corticosterone levels. Blood samples were centrifuged for 15 min at 14,000 RPM, 4 °C to isolate the blood plasma that was frozen and stored. Corticosterone concentrations were further measured with a high-sensitive corticosterone enzyme immunoassay (IDS Ltd, Boldon Colliery, UK).

### 2.4 Tissue collection and processing

Out of 15 Ctrl and 18 ES male mice subject to the behavioral tests described above, 8 Ctrl and 11 ES mice were sacrificed for immunohistochemistry by transcardial perfusion, and their brains were sliced into 40 μm coronal sections in 6 parallel series as described previously (Hoeijmakers et al., 2017; Naninck et al., 2015), while the remaining animals were used for molecular analyses. These 7 Ctrl and 5 ES 20-month-old mice, as well as additional 4- and 10-month (n = 4 per condition) old mice, were sacrificed by rapid decapitation, and their hippocampi were rapidly dissected, frozen and processed for gene expression and relative telomere length analysis. The adrenal and thymus were dissected and weighed, and the skin and internal organs inspected for macroscopic abnormalities as an indicator of general health.

RNA was extracted from the hippocampi using the TRIzol method (TRIzol, Invitrogen). Reverse transcription of RNA to cDNA was performed using SuperScript® III Reverse Transcriptase (Invitrogen). Samples were stored at −20 °C. DNA was extracted from the TRIzol interphase, based on manufacturer's instructions. Briefly, DNA was precipitated using 100% ethanol, washed 3× in 0.1 M sodium citrate dissolved in 10% ethanol (pH 8.5), and eluted in nuclease free water.

### 2.5 Immunohistochemical staining for doublecortin (DCX) and quantification

#### 2.5.1 DCX immunostaining

To study adult neurogenesis, we stained for doublecortin (DCX), which is expressed by newborn cells from a few days after their birth until their early adult neuronal stage. Free-floating sections were washed in 0.05 M TBS (pH 7.6) prior to immunostaining and in-between all incubation steps. After initial washing, peroxidase activity was quenched by incubating in 0.3% H2O2 in TBS for 15 min. Slices were incubated in a blocking mix of 2% milk in TBS for 30 min, and then incubated in primary antibody (1:1600 goat anti-DCX, Santa Cruz Biotechnology, USA) diluted in Supermix (0.1% Triton X-100, 0.25% gelatin in TBS) for 1 h at room temperature, and overnight at 4 °C. Slices were then incubated for 2 h with secondary antibody (1:500 biotinylated donkey anti-goat, Jackson Laboratories, USA) in Supermix, and then incubated for 90 min in an avidin-biotin complex (ABC, 1:800 in TBS, Vectastain Elite ABC-peroxidase kit, Brunschwig Chemie, Netherlands). Signal was amplified by a 30-min incubation with 1:500 biotinylated tyramide in a 0.01% H2O2 TBS solution, followed by a second incubation in ABC for 90 min. Finally, sections were washed in 0.05 M TB (pH 7.6) and incubated in 0.2 mg/ml diaminobenzidine (DAB), 0.01% H2O2 in 0.05 M TB for chromogen development. Free-floating sections were thoroughly washed afterwards in TBS and mounted in 0.01 M PB (pH 7.4) on pre-coated glass slides (Superfrost Plus slides, Braunschweig, Germany). Sections were counterstained with hematoxylin and coverslipped.

#### 2.5.2 DCX quantification

For quantification, DCX+ cells were counted across 8 coronal, bilateral sections throughout the hippocampus on a Zeiss Axiophot light microscope (40× objective) with a Microfire camera using StereoInvestigator software (MBF Bioscience, Williston, VT, USA). 4 sections rostral and 4 sections caudal to bregma −2.30 mm (i.e. ± 300 μm intersection distance) were analyzed to obtain an even representation of the hippocampus over the rostral-caudal axis. All quantification procedures were performed by a researcher blind to the experimental conditions.

DCX-immunoreactive cell numbers in the granular cell layer (GCL) and subgranular zone (SGZ) of the hippocampus were quantified manually on a Nikon Eclipse Ni-E light microscope equipped with a Nikon DS-Ri2 Camera (Nikon, Japan). DCX+ cells were classified into three types based on morphology to reflect their relative maturity, namely 1) Proliferative, or horizontal cells without a process, 2) Intermediate, or cells with an apical process into the GCL, and 3) Postmitotic, or cells with a dendritic tree reaching the molecular layer (Hoeijmakers et al., 2018). The Cavalieri principle was used in order to estimate GCL, SGZ, and DG volume as previously described (Hoeijmakers et al, 2017, 2018b), with the GCL and SGZ volume used to calculate DCX+ cell density.

### 2.6 Gene expression measurement with qRT-PCR

Gene expression analysis was performed as previously described (Hoeijmakers et al., 2017). Briefly, relative gene expression of microglial genes was measured by amplification of cDNA using the Hot FirePol Evagreen qPCR supermix (Solis Biodyne, Estonia). Primer sequences are listed in Supplementary Table 1.

All primer pairs were tested for efficient amplification (between 90 and 110%). Relative gene expression was quantified using the 2^ΔΔct^ method after normalization to 3 reference genes that satisfied the requirements for reference target stability as calculated using qBASE software (Biogazelle, Belgium).

### 2.7 Relative telomere length measurement

Hippocampal telomere length was measured using the qPCR-based T/S, method, which compares relative telomere length by dividing telomeric amplicons by those from a single copy gene (SCG) (Cawthon, 2002). Primers and protocols were adapted from O'Callaghan et al. (2008). Primer sequences (5’→3′) are as follows: Telo fw: CGGTTTGTTTGGGTTTGGGTTTGGGTTTGGGTTTGGGTT, Telo rv: GGCTTGCCTTACCCTTACCCTTACCCTTACCCTTACCCT, SCG fw: GCTTCT-GACACAACTGTGTTCACTAGC, SCG rv: CACCAACTTCATCCACGTTCACC. Data were analyzed using the ΔΔCT method.

### 2.8 Statistical analysis

Data are expressed as the mean ± standard error of the mean (SEM) and were analyzed using R v3.5.1 and graphically visualized using the ggplot2 package (Wickham, 2016). Significant outliers were identified and removed via the 1.5*IQR method. One Ctrl mouse was excluded from all behavioral data analyses. Data were considered statistically significant when p < 0.05.

In this study, multiple mice from the same litters were included in the experimental groups. We tested for litter effects using the *nlme* package in R and included litter as a random factor in a mixed linear model when significant. Single-factor parameters were analyzed with either a Welch-corrected *t*-test, Mann-Whitney *U* test, standard one-way ANOVA, or Welch corrected ANOVA, depending on factor levels and passing of test assumptions, as determined via Levene's test and Shapiro-Wilk test. Analyses with two factors were analyzed using two-way ANOVA. Probe-trial performance was analyzed with the one-sample *t*-test (hypothetical value 25%). Mixed model ANOVAs were used to analyzed MWM data with “acquisition day” as a repeated factor and “condition” as independent factors. Post-hoc analyses were done with Tukey or Games-Howell methods, and adjusted with Bonferroni corrections. Pearson correlation and linear regression analyses were employed to address the contribution of body weight to OF exploration and to MWM escape latency and path length. Equality of variance of CORT data between experimental groups was tested using an F-test.

## 3 Results

### 3.1 Physiological parameters in 20-month-old mice are not affected by ES

ES did not affect the physiological parameters measured at 20 months including body weight, thymus weight, adrenal weight and plasma corticosterone (Fig. 1B); body weight (t(28.53) = 0.5263, p = 0.6028), thymus weight (t(26.00) = −1.55, p = 0.1322), adrenal weight (t(26.15 = −0.3539, p = 0.7263), and corticosterone (peak: t(22.36) = −0.1632, p = 0.8718; trough: U = 115, p = 0.2363; delta: t(22) = −0.7563, p = 0.4575). ES mice also had decreased variance in trough (F(11,14) = 3.1213, p = 0.0484) but not peak (F(11,13) = 0.4096, p = 0.1465) corticosterone levels compared to Ctrl mice (Fig. 1C and D).

### 3.2 ES does not affect behavior in the open field or the water maze

**Fig. 2:**
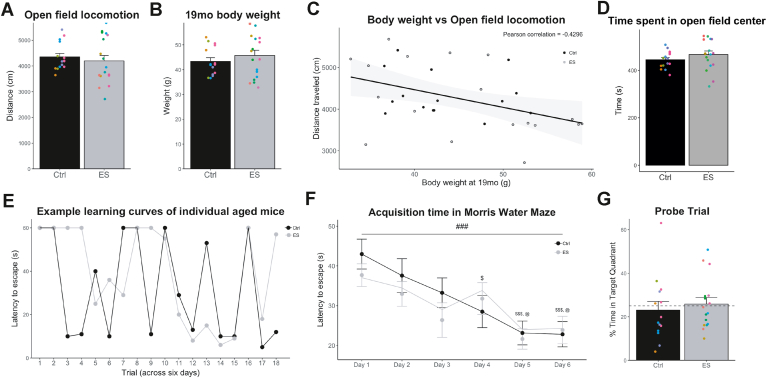
Water maze performance is not further impaired in 19-month-old ES-exposed mice. A) Locomotion in the open field (OF) is not affected by ES. B) Body weight before start of behavioral testing is not different between groups. C) Locomotion in the OF is negatively correlated with the body weight at age of testing. D) Time spent in the center in the OF is also not different between Ctrl and ES mice. E) Example scores of a Ctrl mouse and an ES mouse show the saw-tooth pattern of acquisition typical for aged rodents. F) Both Ctrl and ES mice show a significant decrease in the time to locate the platform over the days of acquisition trainings. G) Time spent in the target quadrant (Tq) during the probe trial is not significant from chance level (dashed line) in both Ctrl and ES mice. Annotations: ###: acquisition day effect, p < 0.001; posthoc: $, different from Day 1, p < 0.05; $$$, different from Day 1, p < 0.001; @, different from Day 2, p < 0.05.

Locomotion behavior (i.e. total distance moved and time spent in center) was measured in the OF in order to examine exploratory behavior, anxiety and possible movement abnormalities. The total distanced moved (t(28.3) = 0.6079, p = 0.5481; Fig. 2A) as well as the velocity (t(28.22) = 0.6211, p = 0.5395; not shown) in the OF were not different between Ctrl and ES mice. Locomotion behavior can be influenced by body weight of the mice, and, while ES did not affect body weight at 19 months of age (t(29.36) = −0.9292, p = 0.3604; Fig. 2B), individual body weight was correlated with the total distance moved (Pearson correlation: r = −0.4296, p = 0.0126; Fig. 2C). The time spent at the center of the OF was not affected by ES (t(27.75) = −1.2615, p = 0.2176; Fig. 2D).

Both groups of mice (Ctrl and ES) exhibited a typical saw-tooth learning pattern in the MWM (Fig. 2E) (Vorhees and Williams, 2006). The average latency to the platform during acquisition did not differ between Ctrl and ES mice, and decreased in both groups over the 6 training days (mixed model ANOVA: condition F(1,28) = 0.82, p = 0.373; time F(5,140) = 8.37, p < 0.001, interaction F(5,140) = 0.691, p = 0.631; post-hoc of time: Day (D) 1 vs D4, p = 0.0378, D1 vs D5, p=<0.001; D1 vs D6, p < 0.001; D2 vs D5, p-0.0390; D2 vs D6, p = 0.0318; Fig. 2F). The average latency over all trials was not different between the Ctrl and ES groups (t(25.1983) = 1.711, p = 0.0994). During the probe trial, neither group of mice performed above chance-level (difference from chance-level (25%), Ctrl: t(13) = -0.484, p = 0.6818; ES: t(15) = 0.284, p = 0.390; Fig. 2G), and there was no difference between conditions (t(25.62) = −1.0933, p = 0.2844). Average swimming speed in the MWM was not different between Ctrl and ES groups (t(28) = 0.05, p = 0.957, not shown).

### 3.3 ES exposure does not affect adult neurogenesis or gene expression of synaptic markers at 20 months of age

**Fig. 3:**
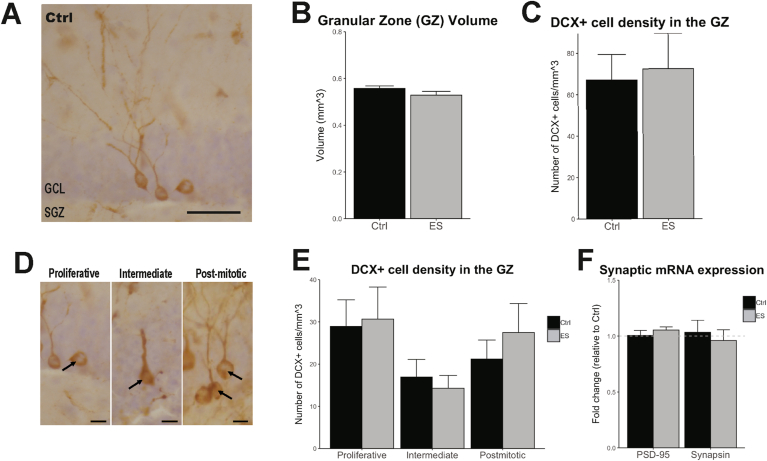
Neuroplasticity in aged mice is not affected by ES. A) Representative image of doublecortin (DCX) immunostaining in the granular cell layer (GCL) and subgranular zone (SGZ) in a 20-month-old Ctrl mouse. B) GZ volume is not affected by ES. C) Total DCX + cell density in the granular zone (GZ, GCL + SGZ) of the hippocampus at 20 months of age is not affected by ES. D) Examples of DCX + cells (arrows) classified as being in a proliferative, intermediate or post-mitotic maturation stage. E) The density of proliferative, intermediate or post-mitotic DCX + cells in the GZ are not changed following ES. F) Synapsin1 and Psd95 mRNA expression in the hippocampus is not different between aged Ctrl and ES mice. Scale bars: A) 50 μm, C) 15 μm.

DCX + cells were counted in the granular cell layer (GCL) and subgranular zone (SGZ) of the dentate gyrus (Fig. 3A), whose combined volume (granular zone, GZ) was not affected by ES (t(12.072) = 1.6904, p = 0.1166; Fig. 3B). Total dentate gyrus volume was also not affected by ES (t(13.543) = 1.608, p = 0.1323, not shown). ES did not change the density of total DCX+ cells in the GZ (t(15.45) = −0.255, p = 0.802, Fig. 3C). When classified based on morphology (Fig. 3D), there was no ES effect on the density of each DCX subtype (Proliferative: t(15.94) = −0.176, p = 0.8628; Intermediate: t(13.41) = 0.513, p = 0.616; Postmitotic: t(14.90) = −0.768, p = 0.455, Fig. 3E). mRNA expression of the synaptic markers synapsin1 and PSD95 was also not affected by ES at this age (Synapsin1 t(9.7418) = 0.4098, p = 0.6908; Psd95 t(8.8694) = −1.209, p = 0.3307; Fig. 3F).

### 3.4 ES exposure does not aggravate aging-associated changes to hippocampal inflammatory gene expression and telomere length

**Fig. 4:**
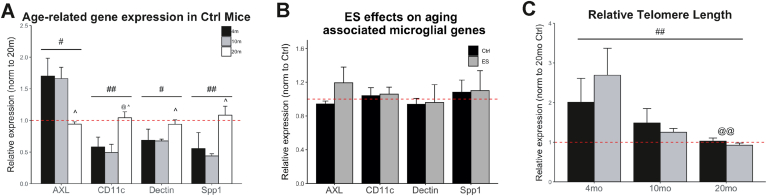
Aging alters hippocampal inflammatory gene expression and relative telomere length without further modulation by ES. A) Hippocampal mRNA expression of CD11b, Dectin, and SPP1 is upregulated in 20-month-old mice compared to the 4 and 10 months age groups, while AXL is downregulated. B) Early-life stress does not lead to differential expression of these four genes at 20 months. C) Relative hippocampal telomere length decreases with aging, but is not affected by early-life stress. Annotations: #, age effect, P < 0.05; ## age effect, p < 0.01; post-hoc: @, significant from 4mo, p < 0.05; @@, significant from 4 months group; ^, different from 10 m, p < 0.05.

We investigated the gene expression of four microglial genes (i.e. Axl, Dectin, Spp1) previously reported to be increased in expression with age in the mouse hippocampus. Aside from AXL, where we found an age-associated decrease, specifically from 10 to 20 months (Welch corrected ANOVA – Axl: F(2,4.6601) = 13.838, p = 0.01096, post-hoc: 4vs10Mo p = 0.9998, 4vs20Mo p = 0.0977, 10vs20Mo p = 0.0189), we confirm the earlier reported, age-associated increase in expression of CD11c, Dectin, and Spp1 in control hippocampus (Welch corrected ANOVA – Dectin: F(2,6.3474) = 6.1731, p = 0.03245; Post-hoc: 4vs10Mo p = 0.9512, 4vs20Mo p = 0.4168, 10vs20Mo p = 0.0107; One-way ANOVA – CD11c: F(2,15) = 7.4399, p = 0.0057, post-hoc: 4vs10 p = 0.88, 4vs20Mo p = 0.0271, 10vs20 p = 0.0169; Spp1 F(2,16) = 7.3789, p = 0.005362, post-hoc: 4vs10Mo p = 0.999, 4vs20 p = 0.0182, 10vs20 p = 0.0202). The expression of these genes was not affected in 20-month-old ES-exposed mice (Axl: t(4.82) = −0.389, p = 0.714; CD11c: t(7.98) = 0.077, p = 0.941; Dectin: t(3.51) = 0.321, p = 0.766; Spp1: t(4.34) = -0.008, p = 0.994; Fig. 4B).

Relative telomere length was decreased in the hippocampi of 20-month-old mice compared to younger age groups, but was not affected by ES at any of the ages studied (condition: F(1) = 1.869, p = 0.18483; age: F(2) = 9.346, p = 0.00107; interaction: F(2) = 0.747, p = 0.4851; post-hoc: 4vs10Mo p = 0.073, 4vs20Mo p = 0.001, 10vs20Mo p = 0.325; Fig. 4C).

## 4 Discussion

We show here that exposure to chronic ES from P2 to P9 did not affect general physiological measures, except that it decreased the variability in basal corticosterone levels compared to controls. ES also did not alter behavioral performance at the age of 20 months. The hippocampi of the ES-exposed mice at this age did not exhibit significant alterations to DG volume, DCX + cell numbers, levels of synaptic and neuroinflammatory gene expression, or relative telomere length. Placing our data in the context with those from previous ES studies on these domains at earlier ages (i.e. at 4 and 10 months) by our group and others, a picture emerges as to how ES alters the aging trajectory.

### 4.1 Cognitive performance in aged rodents with aging

While the consequences of ES for cognition in advanced-aged rodents has been addressed before using ES models in rats (Brunson et al., 2005; Jauregui-Huerta et al., 2015; Lemaire et al., 2000; Oitzl et al., 2000; Schaaf et al., 2001; Solas et al., 2010; Vallée et al., 1999), we present here for the first time, effects of the limited nesting and bedding paradigm in aged mice.

We showed that 19-month-old ES mice did not differ in performance compared to controls. Previous work from our lab and others studying the same model found that, at 4 months, ES-exposed mice exhibit deficits in the MWM and other cognitive tasks (e.g. object recognition) (Naninck et al., 2015; Wang et al., 2011). This is no longer the case by 10 months, when ES-exposed mice perform similarly to Ctrls in the MWM (Hoeijmakers et al., 2018a). Together with data from the current study, this suggests that, while ES mice exhibit an initial cognitive decline in early adulthood, they eventually perform similarly to controls at later ages. This might reflect shared mechanisms between ES and aging, or, as control mice also decline with age, it could also indicate a natural aging-induced floor effect at which any further cognitive impairment is hard to detect (Koh et al., 2014; Lindner, 1997). When interpreting these data, one should bear in mind that the MWM can be somewhat stressful (Enthoven et al., 2008; Schaaf et al., 1999), and that this stressful nature of the learning paradigm could have influenced the observed outcome. Of course, several additional elements could have also impacted the MWM outcome, such as the water temperature (Akirav et al., 2004; Salehi et al., 2010) and the aging-associated loss of visual acuity (Wong and Brown, 2007). However, based on the fact that all included mice acquired the tasks as expected, we do not have reason to believe these were significant confounders in the behavioral paradigm performed.

Lastly, while some studies have found ES exposure via maternal separation or deprivation (MD) to worsen cognitive performance in 20–24-month-old rats (Solas et al., 2010; Vallée et al., 1999), others have found ES to lead to stronger variations in MWM performance in 22–24-month-old rats (Bizon et al., 2009; Oitzl et al., 2000). In fact, Oitzl et al. reported that while average performance was not different in aged MD vs control rats, ES effects became apparent upon taking the distribution of individual performance within each group into account (Oitzl et al., 2000). Our cohort, while powered for a group-level analysis, did not allow for a reliable study of individual variation at the level of behavior. Nonetheless, while the average basal CORT levels in both groups falls into a similar range as those previously reported in young adult mice (Bonapersona et al., 2019; Naninck et al., 2015), the variance in 20-month ES basal CORT levels was lower versus controls, suggesting that individual differences exist within these groups. This will need to be further addressed in future studies.

### 4.2 Chronic ES does not alter doublecortin (DCX) expression and synaptic markers in aged mice

ES exposure has been shown to alter adult neurogenesis, and lead to structural hippocampal changes as evidenced by reduced synaptic protein levels, spine numbers and neurogenic cell survival in ES mice at 3–4 months (Liu et al., 2016; Naninck et al., 2015, Hoeijmakers et al., 2018a). We here studied DCX + cell numbers and *Synapsin1* and *Psd9*5 mRNA expression at 20 months of age and found that none of these measures was affected by ES at this age.

Hippocampal neurogenesis and DCX expression decline with age (Heine et al., 2004; Hoeijmakers et al., 2018b; Kuhn et al., 1996; Seki and Arai, 1995), which is also evident when comparing our data to DCX+ cell numbers from previous cohorts of ES mice at 4 and 10 months from our lab (Naninck et al., 2015; Hoeijmakers et al., 2018a). While ES did not affect total DCX+ cell numbers in the dentate gyrus at 4 months of age, it did reduce the number of immature DCX+ cells in the SGZ subregion at 10 months of age.

Next to DCX, ES effects on neurogenesis are detected in the reduced survival of newborn cells in the hippocampus, as quantified by BrdU, an effect that is most striking around 4–6 months (Kanatsou et al., 2017; Naninck et al., 2015), but not seen at 10 months (Hoeijmakers et al., 2018a). We were not able to reliably address this in the current study, due to the very limited amount of newborn cells at 20 months of age, in contrast to the 2-week-long expression window of DCX which allows for more robust quantification (Brown et al., 2003; Kempermann et al., 2004). Taken together, this would suggest that ES leads to an earlier reduction in neurogenesis, specifically the survival of newborn neurons, which did not further worsen at a more advanced age.

We here measured for the first time mRNA levels of the synaptic markers synapsin-1 and PSD-95 in 20-month-old mice. While we did not detect any differences in relative gene expression of these synaptic markers at this age, brains of pups analyzed immediately after ES have been reported to exhibit deficient dendritic and spine growth, along with decreased levels of synaptic proteins such as synaptophysin, PSD-95, nectins, and NMDA receptors (Liao et al., 2014). Notably, these ES effects persisted until at least P90 (Liu et al., 2016). The synapses also change functionally, with ES decreasing hippocampal LTP at 6 months of age, although protein levels of PSD-95 and synaptophysin were similar in both groups at this age (Lesuis et al., 2019). Importantly, the latter study demonstrates the possibility that functional changes can be observed without parallel changes in absolute protein levels.

Taken together, this suggests ES induced premature deficits at the synapse, which might not be further aggravated at 20 months. Because our current study assessed only gene expression of synaptic markers, further investigations are needed to elaborate on the functional consequences of ES for the aged hippocampal synapse.

### 4.3 The age-induced inflammatory profile is not altered by ES exposure

We further found age-related changes in the expression of Axl, CD11b, Dectin, and Spp1, confirming earlier reports on age-induced alterations in these inflammatory genes (Raj et al., 2015), which are not further altered by ES. Aging is known to induce a pro-inflammatory phenotype, manifesting as microglial priming in the brain (Holtman et al., 2015; von Bernhardi et al., 2015). Indeed, early-life experiences are thought to “prime” the neuroimmune system, which is thought to lead to a stronger microglial response to later challenges (Elwenspoek et al., 2017; Korosi et al., 2015). For example, early-life infections strengthen the age-related pro-inflammatory changes in rats (Bilbo, 2010; Bilbo and Schwarz, 2009), where aging can be thought of as a secondary inflammatory ‘hit’. Similarly, ES leads to an altered neuroinflammatory profile early in development (Delpech et al., 2016; Hoeijmakers et al., 2017; Ślusarczyk et al., 2015) that is still evident at 4 and 10 months, both in basal conditions as well as in response to the progressive amyloid-beta accumulation in a transgenic mouse model (Hoeijmakers et al., 2017).

Our current data suggest that ES does not modulate the aging-associated neuroimmune gene expression per se under basal conditions. Whether changes could be detected at other level of microglia analyses, or if aged ES microglia would respond differently to additional immune challenges, remains to be clarified. Nonetheless, the currently existing evidence suggests that ES leads to premature changes in the neuroimmune profile, which is not further exacerbated by aging.

### 4.4 Hippocampal telomere length decreases with age but is not modulated by ES

Lastly, we found aging itself, but not ES, to have effects on the relative telomere length in the hippocampus, specifically at 20 months. While telomere attrition is one of the best studied hallmarks of aging, only few studies have investigated this in the rodent brain. These include studies on telomere length in the cerebellum and cortex of 5-month-old rats (Flanary and Streit, 2003), as well as in cortical neurons of 25–27-month-old mice (Ain et al., 2018). Our study is to our knowledge the first showing that telomere length also decreases with age in the mouse hippocampus.

Telomere length, mostly measured in leukocytes or saliva, negatively correlates with exposure to early-life stress in humans (reviewed in Shalev et al., 2013). A meta-analysis found this effect to be stronger depending on severity of the stressor, as well as on the time since stress exposure (Ridout et al., 2018). Importantly, these are all measured in non-brain tissues, which is relevant given that aging effects on telomere length can be tissue specific (Coviello-McLaughlin and Prowse, 1997; Prowse and Greider, 1995).

We did not find any ES effects on telomere length at 4, 10 or 20 months. The incongruence here might be due to differences in human and mouse telomere biology, among the most relevant of which might be the difference in lifespan (Calado and Dumitriu, 2013). It could also be a consequence of using whole hippocampal tissue (i.e. a heterogeneous population of cells), as different types of cells vary in their proliferative capacity, and thus telomere lengths (Flanary et al., 2007). Further studies on cell-sorted samples might better elucidate any possible link between ES and telomeres.

### 4.5 Working hypothesis on how ES affects the aging trajectory

**Fig. 5:**
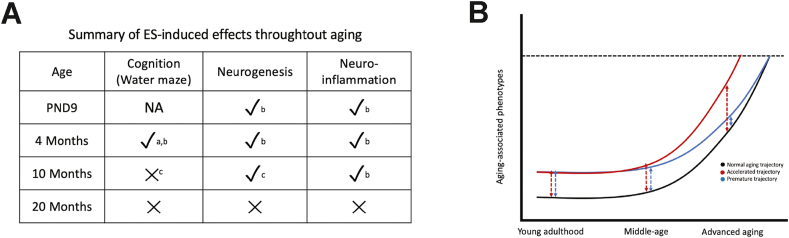
ES leads to premature alterations to aging-associated domains that disappear with advanced aging. A) Comparing ES effects on the aging-associated domains of cognition, neurogenesis and neuroinflammation across several cohorts. P9, 4-month and 10-month data based on previously published results, 20-month based on current study. (Citations: a – Naninck et al., 2015, b – Hoeijmakers et al., 2017, c – Hoeijmakers et al., 2018a). B) Our cumulative data suggest that while ES does not lead to a lasting, progressive shift in the aging trajectory, which would aggravate the aging phenotype (red curve, “accelerated aging”), it instead leads to an early shift in the curve that, while altering the overall trajectory across the lifespan, results in comparable phenotypes by 20 months of age (blue curve, “premature aging”). (For interpretation of the references to colour in this figure legend, the reader is referred to the Web version of this article.)

We have attempted in this discussion to integrate data from this and previous cohorts (Hoeijmakers et al, 2017, 2018a; Naninck et al., 2015) into a more complete understanding of the effects of ES on the aging trajectory ranging from 4, 10, and 20 months of age. While the alterations induced by ES to the domains of cognition, neurogenesis and neuroinflammation (especially at 4 months) have led many to hypothesize a link between ES and accelerated aging, the novel picture that now emerges is that while ES leads to ‘early’ effects on domains also affected by the aging process. However, based on our current data, these domains do not progressively worsen at more advanced ages (Fig. 5A).

There are several explanations for these observations: i) control mice gradually worsening with aging, ii) ES effects disappearing over time, or iii) a ‘floor’ effect beyond which ES mice do not further impair, which is pertinent given how, in mice, 20 months marks the onset of age-related deficits and mortality (Flurkey et al., 2007; Forster et al., 2003). Assuming that ES effects at an earlier age (4 months) indeed shift the aging-associated progression of our measured domains across the lifespan, their trajectories could theoretically be altered either by leading to further alterations that ultimately result in an accelerated and aggravated aging phenotype (Fig. 5B, “accelerated-trajectory”), or, as it seems to be the case based on our data, leading to a set of premature changes which, ultimately, do not surpass that of the typical aged individual (Fig. 5B, “premature trajectory”). Of course, these data do not preclude the possibility that ES might have modulatory effects at in-between as well as at later time-points, or how ES might interact with secondary “hits” that could alter the aging process. We and others have described how ES phenotypes are affected by challenges such as acute stress (Bonapersona et al., 2019), diet (Abbink et al., 2020b; Ruigrok et al., 2021; Yam et al., 2017), and amyloid-β neuropathology (Abbink et al., 2020a; Hoeijmakers et al, 2017, 2018a), and it remains to be seen how these second hits might further alter the aging trajectory in the context of ES.

How ES impact on aging trajectory and aging-associated cognitive decline remains complex. For instance, several discrepancies emerge when studying these effects across species. Rats exposed to the same ES paradigm as the one used in the current study exhibit cognitive deficits in the MWM and other spatial learning tasks progressively with age, being absent in early adulthood (4 months) but emerging by 12 months (Brunson et al., 2005). The paradigm used to test learning can impact the observed effects as well. For example, while object recognition memory progresses with aging in a similar manner as observed in the MWM, object location memory deficits appeared to already be present in 4-month-old ES rats, which persisted until 12 months of age (Short et al., 2020). However, how rats exposed to this ES model perform in these cognitive tasks at advanced ages remains to be determined. In human studies, while early-life experiences can lead to cognitive decline in middle age that lasts until 60–70 years of age, the trajectory it takes is confounded by a variety of social, developmental, and environmental factors, which makes studying ES effects in advanced aging more challenging (Short and Baram, 2019). In addition, the aging process has been shown to differ between sexes (Armstrong et al., 2019), which our study is unfortunately not able to address. Given that there is also evidence for differential responses to ES in male and female mice (Abbink et al., 2017; Naninck et al., 2015), it will be important to also study the how ES affects aging in female mice in future cohorts.

## 5 Conclusion

We studied here the effects of ES exposure on various aging-associated phenotypes. This is the first study to describe the consequences of ES in aged mice. We did not find ES effects in age-related alterations in neuroplasticity, inflammatory markers and relative telomere length. New questions arising from these findings highlight the need to better understand the exact age-related consequences of ES for brain functioning in future studies.

## Funding

AK is funded by 10.13039/501100003246NWO Food cognition and behavior, JPI NutriCog and Alzheimer Nederland; PJL is funded by 10.13039/501100010969Alzheimer Nederland and the Center for Urban Mental Health from the 10.13039/501100001827University of Amsterdam.

## Author contribution

Aniko Korosi: Conceptualization, Writing – original draft, Writing – review & editing. Janssen M. Kotah: Conceptualization, Formal analysis, Methodology, Writing – original draft, Writing – review & editing. Lianne Hoeijmakers: Conceptualization, Formal analysis, Methodology, Writing – original draft. Erik Nutma: Formal analysis, Methodology. Paul J. Lucassen: Writing – review & editing, All authors have read and agreed to the published version of the manuscript.

## Declaration of competing interest

The authors declare no conflicts of interest.

## Data Availability

Data will be made available on request.
