# Supplementary material for: Early-life stress does not alter spatial memory performance, hippocampal neurogenesis, neuroinflammation, or telomere length in 20-month-old male mice
Source: Neurobiol Stress. 2021 Aug 12;15:100379. doi: 10.1016/j.ynstr.2021.100379 (PMC8369064; doi:10.1016/j.ynstr.2021.100379)
Supplement: Multimedia component 1 [file mmc1.docx]

**Supplementary Table 1. Primer sequences used for RT-qPCR**

| **Gene** | **Forward** | **Reverse** |  |
| --- | --- | --- | --- |
| Reference genes | | | |
| Rpl13a | 5’CCCTCCACCCTATGACAAGA3’ | 5’TCGCCTGTTTCCGTAACCTC3’ |  |
| Rpl0 | 5’GCTTCATTGTGGGAGCAGACA3’ | 5’CATGGTGTTCTTGCCCATCAG3’ |  |
| Sdha | 5’GTTGCTGTGTGGCTGACTG3’ | 5’GCACAGTGCAATGACACCAC3’ |  |
| Target genes | | | |
| Synapsin | 5’CAGCACAACATACCCTGTGG3’ | 5’GGTCTTCCAGTTACCCGACA3’ |  |
| Psd-95 | 5’GTACCTAAAGGTGGCCAAGC3’ | 5’CTCATTGTCCAGGTGCTGAG3’ |  |
| Axl | 5’AGACGATGGGGTGGGTATCT3’ | 5’GAAGGAGCTTTTCCAGCCGA3’ |  |
| CD11c | 5’AGTGTCGTATTTGGCTTCCCA3’ | 5’CACGGGGTAGAACAGAGTGA3’ |  |
| Dectin | 5’AAAGCCAAACATCGTCTCACC3’ | 5’GGCCCTTCACTCTGATTGCG3’ |  |
| Spp1 | 5’TTCCAATGAAAGCCATGACCAC3’ | 5’CGACTGTAGGGACGATTGGAG3’ |  |
